# Supplementary figures and images for: Shade Avoidance and Light Foraging of a Clonal Woody Species, Pachysandra terminalis
Source: Plants (Basel). 2021 Apr 20;10(4):809. doi: 10.3390/plants10040809 (PMC8074284; doi:10.3390/plants10040809)

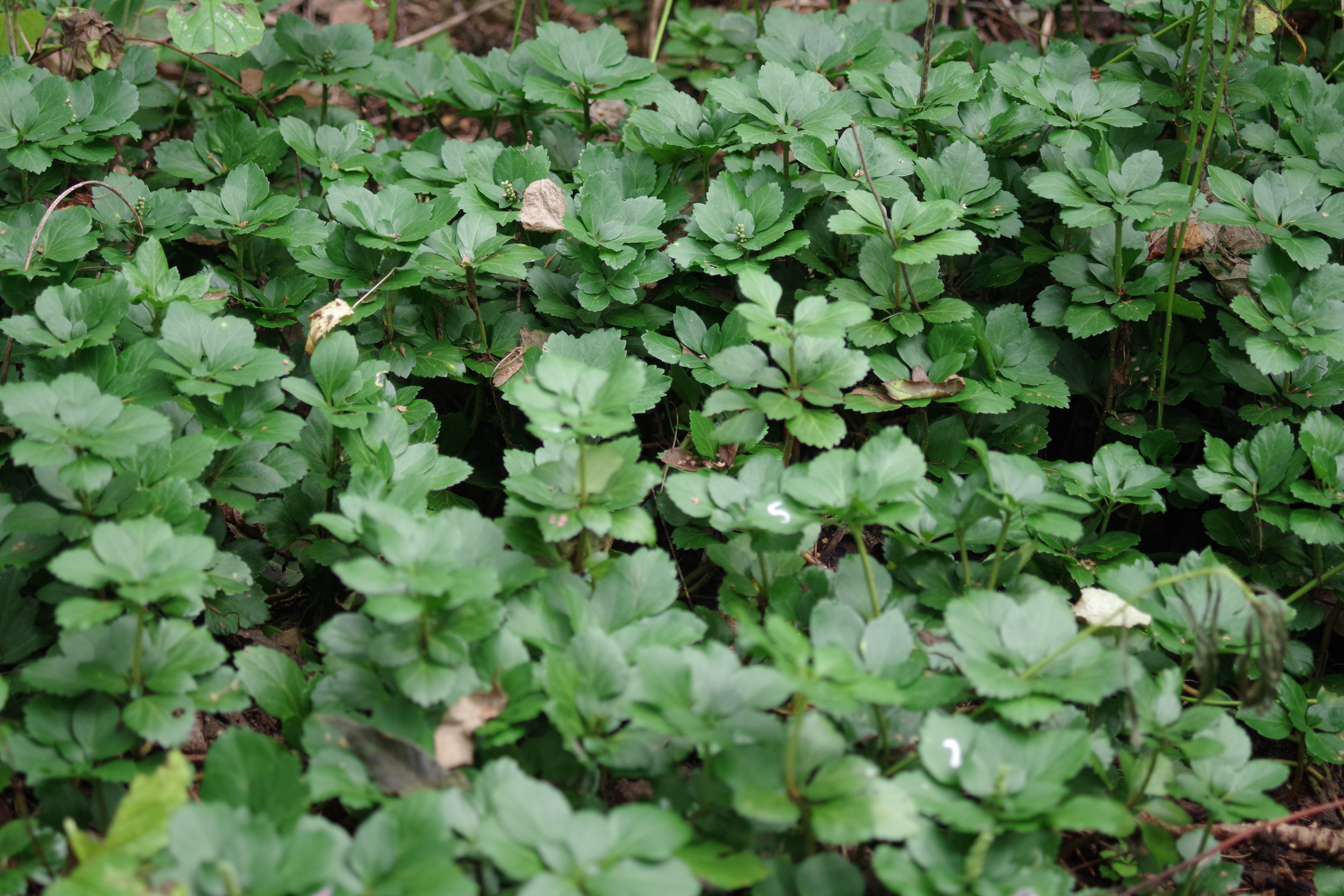

Supplement: Supplementary file 1 [file plants-10-00809-s001.zip › pictures/dense_04_01_koyama_2020_1001_IMGP8854.JPG]

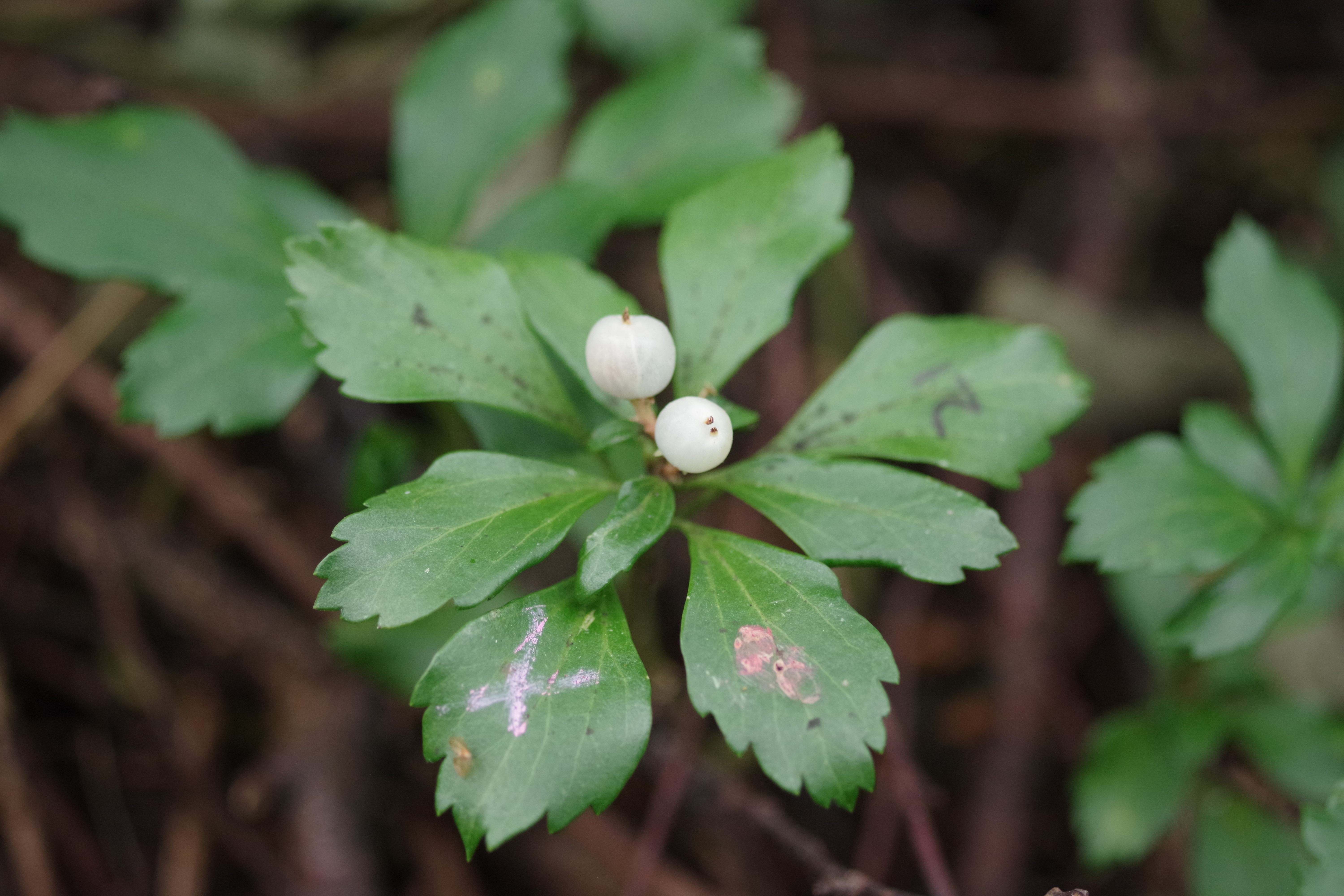

Supplement: Supplementary file 1 [file plants-10-00809-s001.zip › pictures/fruits_koyama2020_1001_IMGP8801.JPG]

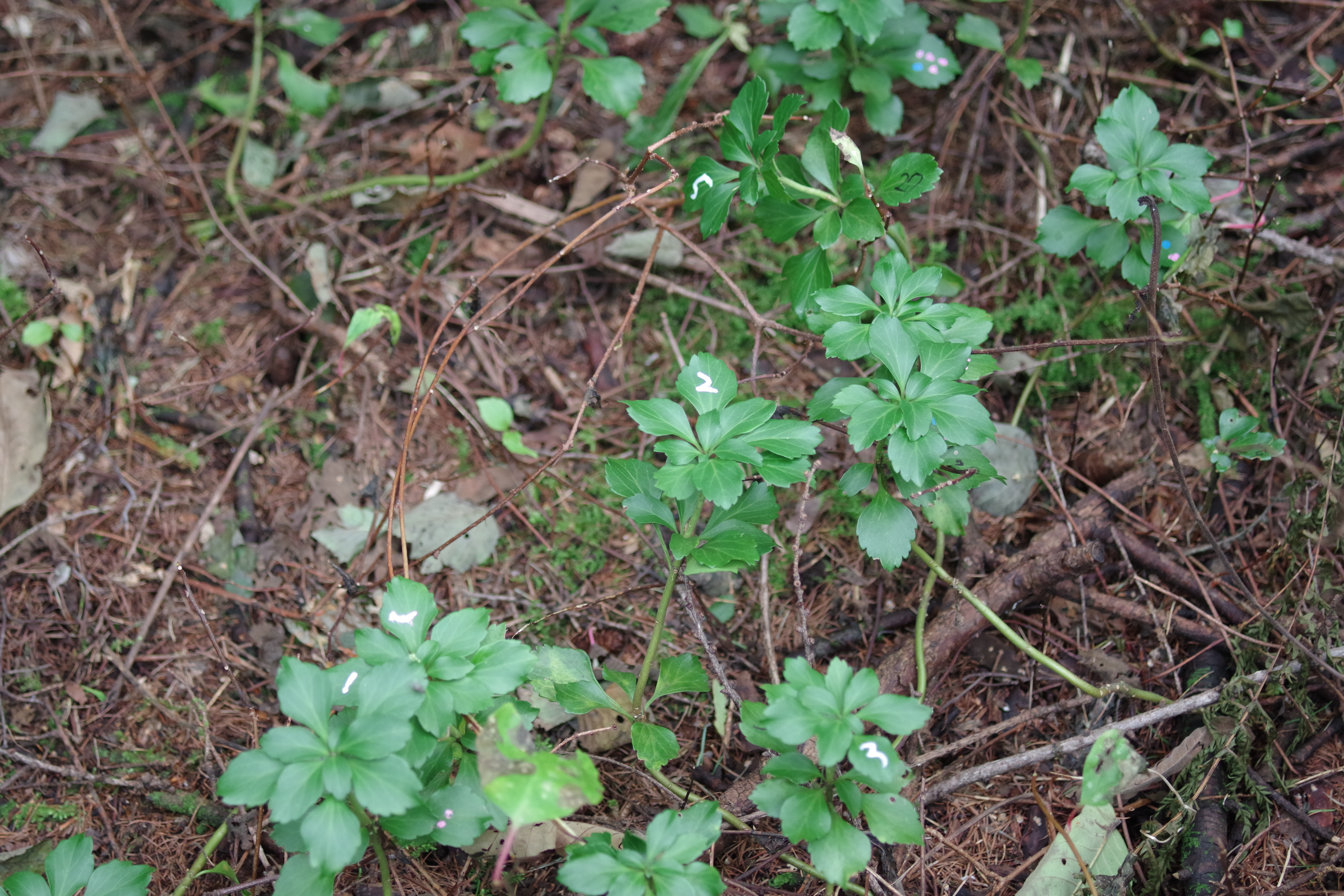

Supplement: Supplementary file 1 [file plants-10-00809-s001.zip › pictures/sparse_09_01_koyama2020_1001_IMGP8839.JPG]

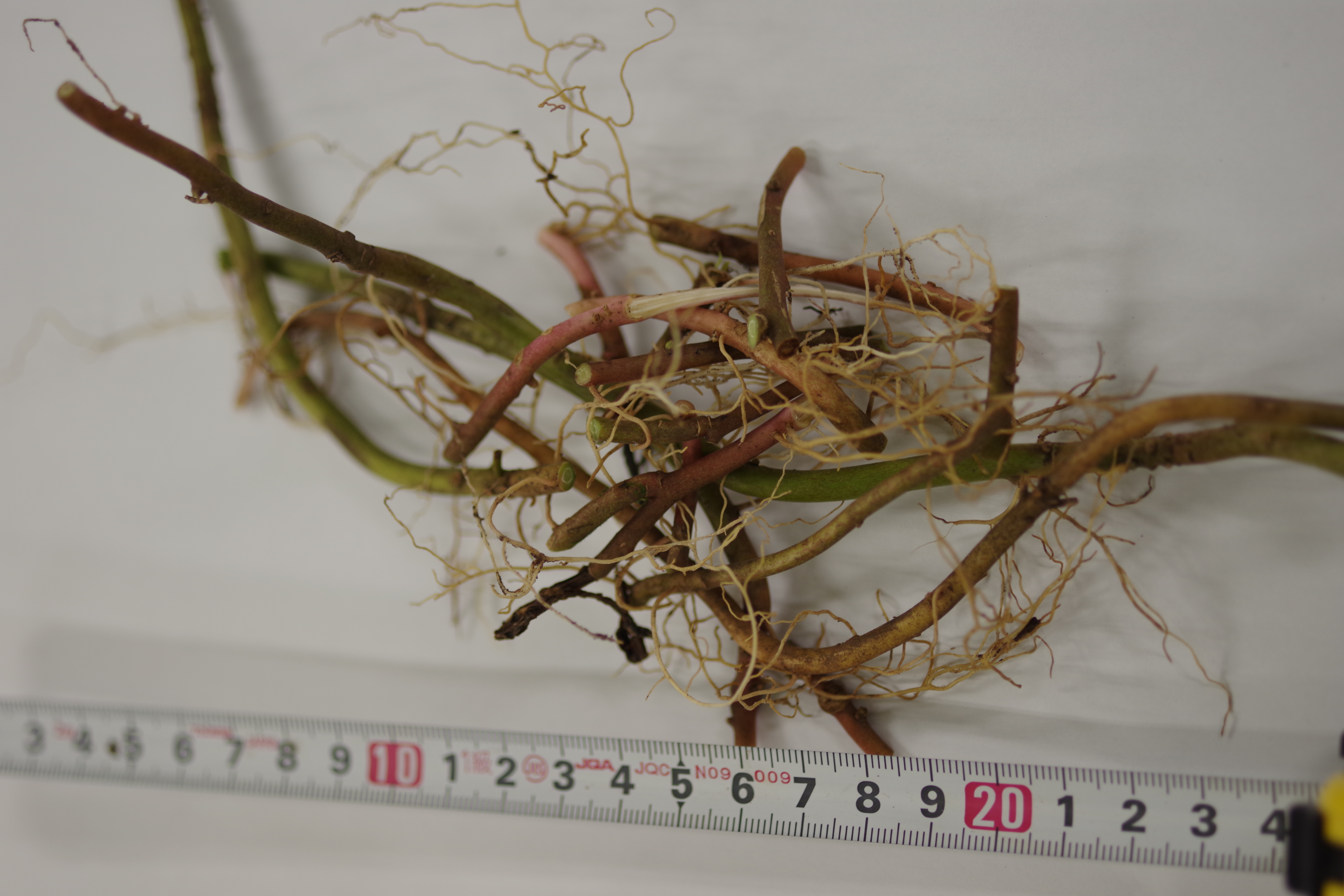

Supplement: Supplementary file 1 [file plants-10-00809-s001.zip › pictures/thick_rhizome_koyama_2020_1018_IMGP8929.JPG]

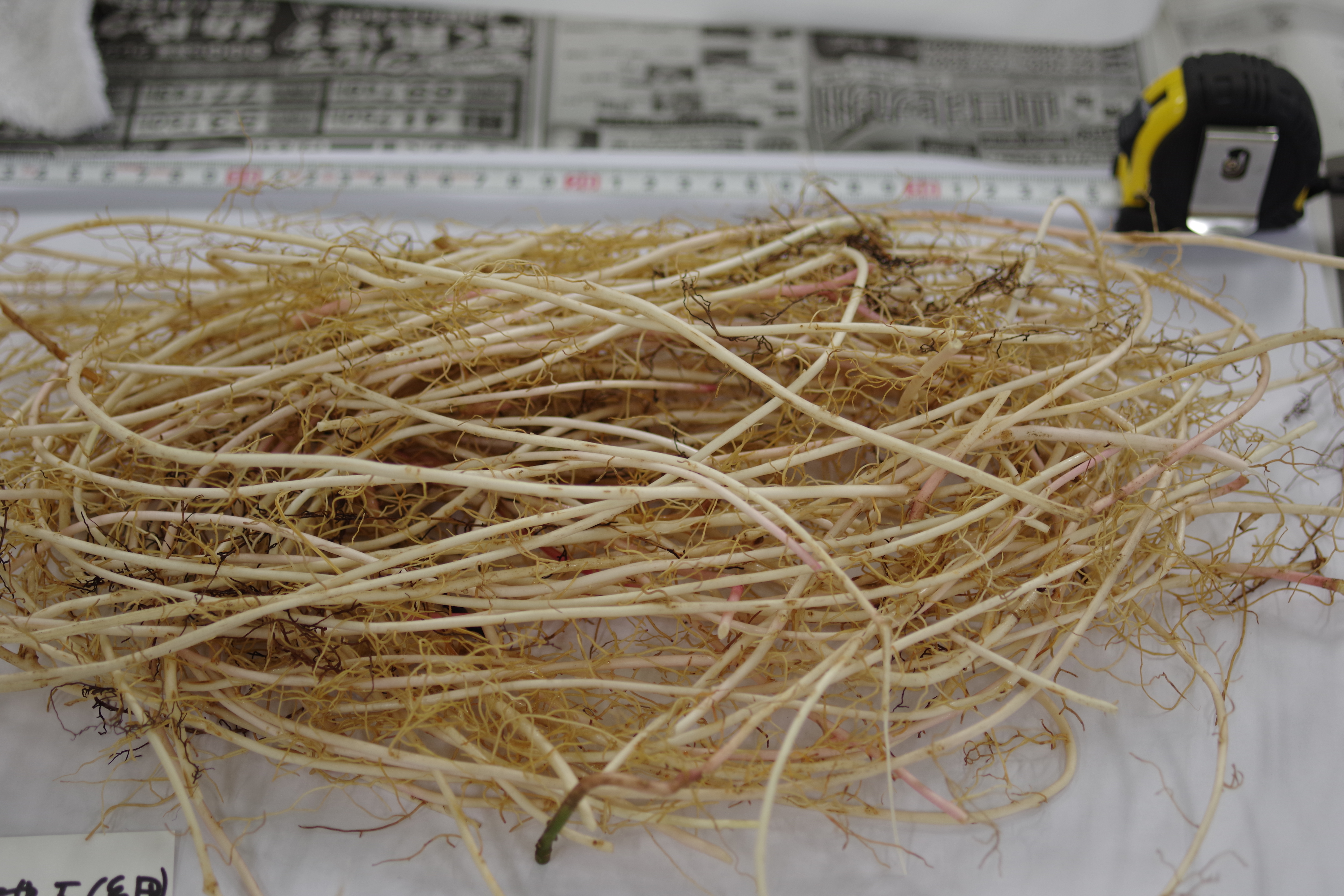

Supplement: Supplementary file 1 [file plants-10-00809-s001.zip › pictures/thin_rhizome_koyama_2020_1018_IMGP8933.JPG]
